# The Roles of YouTube and WhatsApp in Dementia Education for the Older Chinese American Population: Longitudinal Analysis

**DOI:** 10.2196/18179

**Published:** 2020-04-13

**Authors:** Sara Shu, Benjamin K P Woo

**Affiliations:** 1 University of California, Los Angeles Sylmar, CA United States; 2 College of Osteopathic Medicine of the Pacific Western University of Health Sciences Pomona, CA United States

**Keywords:** dementia, mental health, social media, geriatrics, health promotion, health education

## Abstract

**Background:**

Dementia remains a stigmatized topic in the Chinese community.

**Objective:**

This study aims to analyze and compare the usage of dementia educational YouTube videos and the modalities of video sharing over a 6-year period.

**Methods:**

Dementia educational videos were uploaded to YouTube. Data was collected over a 6-year period. Results from the first 3 years were compared to those from the second 3 years using descriptive statistics and chi-square analysis.

**Results:**

Over 6 years, the dementia educational videos generated a total watch time of 269,388 minutes, 37,690 views, and an average view duration of 7.1 minutes. Comparing the first and second 3-year periods of video performance data, there was a longer watch time (59,262 vs 210,126 minutes), more total views (9387 vs 28,303 views), and a longer average view duration (6.3 vs 7.4 minutes). Furthermore, WhatsApp has become a leading external traffic source and top sharing service, accounting for 43.5% (929/2137) and 67.0% (677/1011), respectively.

**Conclusions:**

Over 6 years, YouTube has become an increasingly popular tool to deliver culturally sensitive dementia education to Chinese Americans. WhatsApp continues to be the preferred method of sharing dementia education and has become a top external traffic source to dementia educational videos. Taken together, these social media platforms are promising means of reducing the disparity in dementia knowledge in linguistically and culturally isolated populations.

## Introduction

Social media has become a powerful means for health communication to and among the general public, patients, and health professionals alike [1]. Since its debut in 2005, YouTube has become one of the world’s most popular social media platforms and a major contributor to the accessibility and dissemination of health information around the world [2].

Dementia is a progressive and irreversible neurocognitive disorder that severely impairs an individual’s ability to independently function, which inadvertently causes tremendous burden to family members and caregivers [3]. Although no current therapy can reverse the steady cognitive decline, early and accurate diagnosis has proven efficacious in prolonging quality of life and allowing both the patient and family time to adjust to changes [4]. Consequently, it is necessary to increase public awareness of this diagnosis to promote early intervention amongst all ethnic communities.

Dementia remains a stigmatized subject in the Chinese community, and the scant availability of language-appropriate and culturally sensitive education is a barrier to encouraging those affected from seeking help [5-7]. Work has been done to promote dementia awareness in the Chinese-American community via radio shows, television episodes, and short films [8-10]. Previous studies have demonstrated that YouTube has the potential to successfully deliver dementia education to the older Chinese population [11-15]. In addition, studies have also identified WhatsApp as a promising means for disseminating such dementia education [16].

In this study, we aim to analyze how the Chinese-speaking public search for and share dementia educational videos and evaluate the usage of various social media platforms over time. To our knowledge, this is the first study to present such longitudinal data.

## Methods

### YouTube

A board-certified psychiatrist delivered 2 educational talk shows in Cantonese on a North American Chinese television station in Los Angeles, California. The content of the videos included discussing the background, management, and prevention of dementia. Real-time recordings were subsequently uploaded to YouTube as two 25-minute videos.

### Sample

The sample of this study included YouTube video viewers over a 6-year period (January 2014-December 2019).

### Statistical Analysis

Data was extrapolated from YouTube Analytics. A number of parameters were recorded including number of views, watch time, average view duration, devices used to view, traffic sources, and modes and means of sharing via various social media platforms. Years 1 to 3 (January 2014-December 2016) and years 4 to 6 (January 2017-December 2019) were dichotomized. Descriptive statistics and chi-square tests were used to compare data collected between the first and second 3-year intervals.

This study used anonymous data collected by YouTube exclusively. A waiver for Institutional Review Board exemption was obtained through the University of California, Los Angeles-Human Subjects Protection Committee.

## Results

In 6 years, the two videos of interest accrued a total watch time of 269,388 minutes and 37,690 views, resulting in an average view duration of 7.1 minutes. Broken down into two 3-year intervals, the data are as follows. The latter 3 years had increased performance in all parameters: longer total watch time increased number of views, and longer average view duration.

The average view duration on mobile devices (eg, mobile phone, tablet) between the former and latter 3-year intervals increased 17.0% from 6.9 minutes to 8.1 minutes. On the other hand, average view duration on computers increased 6.9% from 5.8 minutes to 6.2 minutes. There is a significant increase in mobile device usage compared to computer usage from the first 3-year window to the second 3 year window (66.6% vs 78.0%, χ^2^_1_=488.05, *P*<.001) (Table 1).

**Table 1:** Devices used for viewing.

Devices	Jan 2014-Dec 2016			Jan 2017-Dec 2019		
	Minutes	Views	AVD^a^ (min)	Minutes	Views	AVD (min)
Computer	18,292	3132	5.8	38,352	6226	6.2
Mobile device	40,970	6255	6.9	171,774	22,077	8.1
Total	59,262	9387	6.3	210,126	28,303	7.4

^a^AVD: average view duration.

Comparing traffic sources, externally sourced views increased from 6.6% (621/9387 views) to 7.5% (2137/28,303 views) between the two 3-year periods. Of external sources, the top platforms remained Google search and WhatsApp, followed by Facebook. Over 6 years, traffic generated from Google search decreased from 1.5% to 1.1% of total views (138/9387 vs 302/28,303), Facebook generated traffic source decreased from 0.3% to 0.2% of total views (30/9387 vs 54/28,303), and WhatsApp traffic source increased from 0.6% to 3.3% of total views (59/9387 vs 929/28,303). Between the former and latter 3-year periods, WhatsApp traffic source surpassed that of Google search with statistical significance (0.6% vs 3.3%, χ^2^_3_=225.78, *P*<.001) (Table 2).

Table 3 shows the comparison between different sharing services. In comparing the two periods, there was a significant increase in usage of WhatsApp for sharing and movement away from sharing via email (66.4% vs 66.9%, χ^2^_4_=27.82, *P*<.001).

Taken together, although there is a significant increase in traffic generated from WhatsApp between the 2 periods, WhatsApp remains more frequently used for sharing services than viewing capabilities (66.9% vs 3.3%, χ^2^_1_=33.3, *P*<.001).

**Table 2:** Traffic sources.

Traffic sources		Jan 2014-Dec 2016			Jan 2017-Dec 2019		
		Minutes	Views	AVD^a^ (min)	Minutes	Views	AVD (min)
**Top 4 traffic sources**							
	Suggested videos	39,432	5288	6.6	128,448	15,942	8.1
	YouTube search	9468	1696	5.6	8130	1606	5.1
	Browse features	6642	954	6.9	46,680	6345	7.4
	External	3228	621	5.2	11,400	2137	5.3
**Top 4 external traffic sources**							
	Google search	600	138	4.3	1404	302	4.6
	WhatsApp	336	59	5.7	4656	929	5.0
	Facebook	222	30	7.4	378	54	7.0
	Apple app	294	31	9.5	270	30	9.0

^a^AVD: average view duration.

**Table 3:** Sharing services.

Sharing services	Jan 2014-Dec 2016, n=175 (times shared), n (%)	Jan 2017-Dec 2019, n=1011 (times shared), n (%)
WhatsApp	111 (66.4)	677 (67.0)
Email	23 (13.1)	39 (3.8)
Facebook	6 (3.4)	29 (2.8)
Text message	6 (3.4)	60 (5.9)
Other	29 (16.6)	206 (20.4)

## Discussion

### Principal Findings

As the power of social media increases, YouTube has become an indispensable e-mental health platform. In this study, we aimed to investigate the means that the Chinese-speaking public search for and share dementia education videos, and evaluate the usage of various social media platforms over time. Although prior studies have aimed to analyze the potential of YouTube and WhatsApp as means for disseminating dementia education, this is the first study to analyze data over a 6-year period—comparing the first and second 3 years—and provide statistical analysis of not only the trends in devices used for viewing but also the dynamic shift in traffic sources and usage of sharing services [11-16]. Overall, the latter 3 years had increased performance than the first 3 years including longer total watch time (59,262 vs 210,126 minutes), increased number of views (9387 vs 28,303 views), and longer average view duration (6.3 vs 7.4 minutes). Previous studies have reported a 6-minute median engagement time across online educational videos of varying lengths [17]. The increase in average view duration in the second 3-year period from 6.3 to 7.4 minutes demonstrates that the dementia educational videos were able to not only capture but also retain viewers’ attention.

The chi-square analysis showed significant difference in our following analyses. Compared to computer use, average view duration on mobile devices remained and increased far more than on computers between the first and second 3-year intervals (6.9-8.1 minutes). This demonstrates the increasing popularity of using a mobile device over a computer to watch the educational videos. Electronic health (eHealth) communication efforts should therefore ensure mobile device compatibility to maximize audience.

Analysis of traffic sources revealed that YouTube’s suggested videos have consistently been the main source of traffic in the last 6 years. However, the analysis of external traffic sources revealed notable findings. Comparing the first and second 3-year periods, Google search traffic source remained relatively the same (1.5%, 138/9387 to 1.1%, 302/28,303 of views), Facebook generated traffic source decreased (0.3%, 30/9387 to 0.2%, 54/28,303 of views), and WhatsApp traffic source increased and even surpassed that of Google search (0.6%, 59/9387 to 3.3%, 929/28,303 of views). This demonstrates the significant rise in popularity of WhatsApp in the latter 3 years and suggests that WhatsApp could be the future platform for dementia education and eHealth communication alike.

Our study also demonstrates that WhatsApp has evolved into a leading means of sharing dementia knowledge among the Chinese American community. Between the first and second 3-year periods, WhatsApp and text message have both experienced increase in usage as sharing services, while Facebook and email have both dramatically decreased. Previous studies have suggested that WhatsApp is used more for its sharing capability than for its viewing function [16]. Our study confirms this finding and reinforces the claim with 6 years of longitudinal data. Not only has WhatsApp become the preferred means of sharing dementia knowledge, but it has also exceeded Google search and has become the top external source of traffic.

### Limitations

This study has several limitations. First is the lack of demographic information about the viewers. Because all video content was in Chinese and limited to US residents, it was assumed that our study population was largely Chinese American. The ability to collect demographic information would enable analysis of the impact of ethnicity and socioeconomic status on social media use. Second, because there was no measure of viewers’ knowledge of dementia prior to and after watching the videos, we were unable to evaluate the effectiveness of the videos as an educational tool. Third, this study is a retrospective longitudinal analysis of data extrapolated from YouTube Analytics. As such, analysis was constrained to the variables and data collected by YouTube Analytics. In the future, prospective studies should focus on the role of WhatsApp in the dissemination of eHealth content. Last, as each video was 25-minutes long, there was a relatively short average viewing duration of 6.3 to 7.4 minutes. A shorter average viewing duration would preclude effective delivery of important information. Shortening the video length and applying more engaging, interactive content could inherently improve viewer attention, viewing duration, and, ultimately, retention of educational content. Efforts to decrease stigma and negative perceptions toward dementia are essential to provide and coordinate care for the Chinese American population. Future directions include the continued study of the long-term impact that social media has in health communication to populations of interest.

### Conclusion

YouTube has proven to be a valuable tool to deliver culturally sensitive dementia education to Chinese Americans, thereby reducing the disparity of dementia knowledge in linguistically and culturally isolated populations. WhatsApp continues to be a preferred method of sharing dementia education and has become a top external traffic source to dementia educational videos. Use of these findings and continued study of how social media can be used in health communication are imperative to work in disseminating knowledge, reducing stigma, and promoting early detection and treatment in the older Chinese American population.

## Abbreviations

AVD: average view duration
eHealth: electronic health
